# The Gene Ontology resource: enriching a GOld mine

**DOI:** 10.1093/nar/gkaa1113

**Published:** 2020-12-08

**Authors:** The Gene Ontology Consortium, Seth Carbon, Eric Douglass, Benjamin M Good, Deepak R Unni, Nomi L Harris, Christopher J Mungall, Siddartha Basu, Rex L Chisholm, Robert J Dodson, Eric Hartline, Petra Fey, Paul D Thomas, Laurent-Philippe Albou, Dustin Ebert, Michael J Kesling, Huaiyu Mi, Anushya Muruganujan, Xiaosong Huang, Tremayne Mushayahama, Sandra A LaBonte, Deborah A Siegele, Giulia Antonazzo, Helen Attrill, Nick H Brown, Phani Garapati, Steven J Marygold, Vitor Trovisco, Gil dos Santos, Kathleen Falls, Christopher Tabone, Pinglei Zhou, Joshua L Goodman, Victor B Strelets, Jim Thurmond, Penelope Garmiri, Rizwan Ishtiaq, Milagros Rodríguez-López, Marcio L Acencio, Martin Kuiper, Astrid Lægreid, Colin Logie, Ruth C Lovering, Barbara Kramarz, Shirin C C Saverimuttu, Sandra M Pinheiro, Heather Gunn, Renzhi Su, Katherine E Thurlow, Marcus Chibucos, Michelle Giglio, Suvarna Nadendla, James Munro, Rebecca Jackson, Margaret J Duesbury, Noemi Del-Toro, Birgit H M Meldal, Kalpana Paneerselvam, Livia Perfetto, Pablo Porras, Sandra Orchard, Anjali Shrivastava, Hsin-Yu Chang, Robert Daniel Finn, Alexander Lawson Mitchell, Neil David Rawlings, Lorna Richardson, Amaia Sangrador-Vegas, Judith A Blake, Karen R Christie, Mary E Dolan, Harold J Drabkin, David P Hill, Li Ni, Dmitry M Sitnikov, Midori A Harris, Stephen G Oliver, Kim Rutherford, Valerie Wood, Jaqueline Hayles, Jürg Bähler, Elizabeth R Bolton, Jeffery L De Pons, Melinda R Dwinell, G Thomas Hayman, Mary L Kaldunski, Anne E Kwitek, Stanley J F Laulederkind, Cody Plasterer, Marek A Tutaj, Mahima Vedi, Shur-Jen Wang, Peter D’Eustachio, Lisa Matthews, James P Balhoff, Suzi A Aleksander, Michael J Alexander, J Michael Cherry, Stacia R Engel, Felix Gondwe, Kalpana Karra, Stuart R Miyasato, Robert S Nash, Matt Simison, Marek S Skrzypek, Shuai Weng, Edith D Wong, Marc Feuermann, Pascale Gaudet, Anne Morgat, Erica Bakker, Tanya Z Berardini, Leonore Reiser, Shabari Subramaniam, Eva Huala, Cecilia N Arighi, Andrea Auchincloss, Kristian Axelsen, Ghislaine Argoud-Puy, Alex Bateman, Marie-Claude Blatter, Emmanuel Boutet, Emily Bowler, Lionel Breuza, Alan Bridge, Ramona Britto, Hema Bye-A-Jee, Cristina Casals Casas, Elisabeth Coudert, Paul Denny, Anne Estreicher, Maria Livia Famiglietti, George Georghiou, Arnaud Gos, Nadine Gruaz-Gumowski, Emma Hatton-Ellis, Chantal Hulo, Alexandr Ignatchenko, Florence Jungo, Kati Laiho, Philippe Le Mercier, Damien Lieberherr, Antonia Lock, Yvonne Lussi, Alistair MacDougall, Michele Magrane, Maria J Martin, Patrick Masson, Darren A Natale, Nevila Hyka-Nouspikel, Sandra Orchard, Ivo Pedruzzi, Lucille Pourcel, Sylvain Poux, Sangya Pundir, Catherine Rivoire, Elena Speretta, Shyamala Sundaram, Nidhi Tyagi, Kate Warner, Rossana Zaru, Cathy H Wu, Alexander D Diehl, Juancarlos N Chan, Christian Grove, Raymond Y N Lee, Hans-Michael Muller, Daniela Raciti, Kimberly Van Auken, Paul W Sternberg, Matthew Berriman, Michael Paulini, Kevin Howe, Sibyl Gao, Adam Wright, Lincoln Stein, Douglas G Howe, Sabrina Toro, Monte Westerfield, Pankaj Jaiswal, Laurel Cooper, Justin Elser

## Abstract

The Gene Ontology Consortium (GOC) provides the most comprehensive resource currently available for computable knowledge regarding the functions of genes and gene products. Here, we report the advances of the consortium over the past two years. The new GO-CAM annotation framework was notably improved, and we formalized the model with a computational schema to check and validate the rapidly increasing repository of 2838 GO-CAMs. In addition, we describe the impacts of several collaborations to refine GO and report a 10% increase in the number of GO annotations, a 25% increase in annotated gene products, and over 9,400 new scientific articles annotated. As the project matures, we continue our efforts to review older annotations in light of newer findings, and, to maintain consistency with other ontologies. As a result, 20 000 annotations derived from experimental data were reviewed, corresponding to 2.5% of experimental GO annotations. The website (http://geneontology.org) was redesigned for quick access to documentation, downloads and tools. To maintain an accurate resource and support traceability and reproducibility, we have made available a historical archive covering the past 15 years of GO data with a consistent format and file structure for both the ontology and annotations.

## INTRODUCTION

The Gene Ontology (GO) resource is the world's most comprehensive source of information about the function of genes and gene products (proteins and non-coding RNAs). This information is not only human-readable but also machine-readable and therefore plays a critical role in the computational analysis of genomic and biomedical data. GO has been cited by over 100 000 scientific publications to date. The resource is created and maintained by a large consortium of expert biologists, data scientists and software engineers, with invaluable input from collaborating scientists in specific areas of biology. It covers genes from many different organisms from all kingdoms of life, as well as viruses. For well-studied organisms, most of the information in the GO knowledgebase is derived directly from published experiments, while for less well-studied organisms, the information is inferred from sequence homology or from other inference methods. Regardless, a gene function annotated in GO is always supported by specific evidence describing both the source (e.g. PMID of the article) and the type of the evidence from a subset of Evidence and Conclusion Ontology (ECO) (1).

The GO knowledgebase consists of three main components: the GO ontology, the standard GO annotations, and the GO-Causal Activity Models (GO-CAMs) (2). In addition, GO develops tools (see GO toolset) to help users apply the GO knowledgebase in their analyses (3–5). The **GO ontology** provides a formal structure that defines the universe of possible gene functions (GO terms), and the relationships between them (6). **Standard GO annotations** are assertions that relate a specific gene with a specific ontology term describing its function, along with the evidence for that assertion (7). **GO-CAMs** are a relatively new component in the GO resource (2). GO-CAMs link multiple standard GO annotations into a more comprehensive model of gene function and allow causal influences between molecular activities to be detailed using relations from the Relation Ontology (https://github.com/oborel/obo-relations). GO-CAMs currently are of two main types: those that include causal influences between activities (e.g. a biological pathway), and those that do not.

Here we describe the developments in the Gene Ontology resource since our last update article (7). We focus on our progress on the curation and public release of GO-CAM models, the continuous collaborative work to refine the GO ontology, the review and improvement of the quality of our annotations, and the expansion of the usability of GO through a redesigned website, documentation, data access, visualization widget and 15 years of historical archives of the GO knowledgebase.

## CURATED GO-CAMs

### GO-CAM in a nutshell

**Figure 1.**
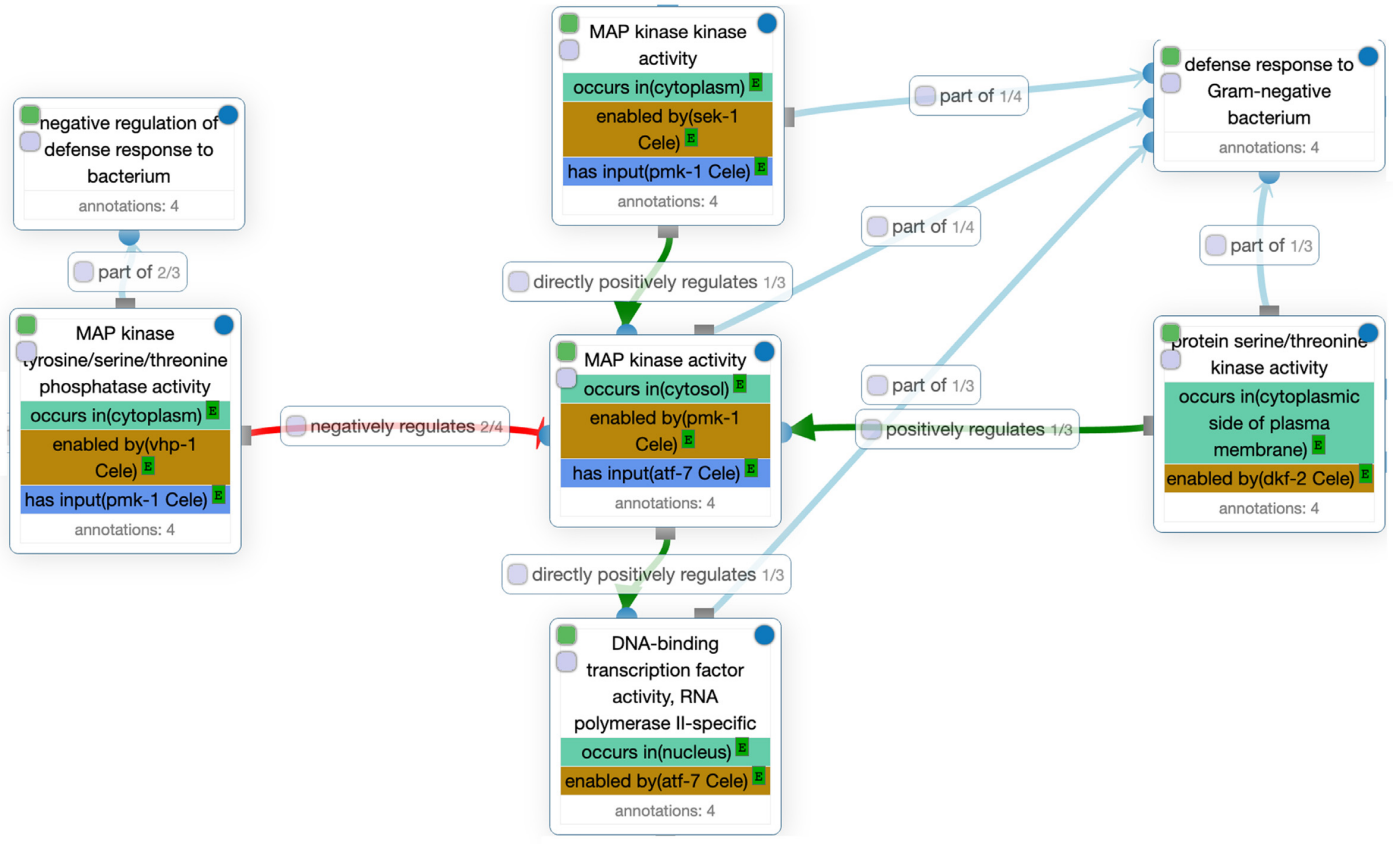
Gene Ontology Causal Activity Model (GO-CAM) of ‘*C. elegans* defense response to Gram negative bacterium in the intestine’. A GO-CAM is centered around at least one molecular activity that takes place within a specific biological context. This part of the GO-CAM details the flow of causal influences between molecular activities with the vocabulary from the Relation Ontology. For instance, the ‘MAP kinase tyrosine/serine/threonine phosphatase activity’ negatively regulates the MAP kinase activity of pmk-1 Cele that occurs in cytosol, which has the downstream effect of negatively regulating the ‘DNA binding transcription factor activity, RNA polymerase II-specific’ of atf-7 cele that occurs in nucleus.

GO-CAM is a strict semantic, descriptive and computable model that enables curators to link together standard GO annotations and enrich them with biological context. In addition, GO-CAMs allow curators to link molecular activities (instances of GO molecular function terms) through causal relationships to describe larger biological programs (Figure 1). We continue to develop Noctua, the new GO curation platform (see GO toolset), to support the curation and review of both standard and GO-CAM annotations. The GO-CAM documentation, data and download are directly accessible through http://geneontology.org/go-cam. As GO-CAMs link standard GO annotations, those GO annotations can be integrated into the GO annotation files used in traditional GO-based analyses like statistical GO enrichment testing.

### Ensure the creation of valid GO-CAMs in Noctua with ShEx

We have implemented a computational schema for GO-CAM using ShEx (Shape Expressions), a formal language for describing structures in Resource Description Format (RDF) graphs (https://shex.io), also used in projects such as Wikidata (https://www.wikidata.org/wiki/Wikidata:WikiProject_Schemas). The use of a computational schema such as ShEx allows Noctua to provide curators with realtime checks and feedback when creating models. It is also used to detect and report errors and inconsistencies in the current repository of GO-CAMs and when migrating standard GO annotations to GO-CAMs (see below). Note that the ShEx schema validation process is an extension of pre-existing logical checks implemented using OWL reasoning (https://doi.org/10.5281/zenodo.2397192). ShEx provides a way to specify ‘closed world’ constraints that aren’t suitable for OWL representation. For example, the schema includes constraints that define allowable evidence and provenance structures for each assertion within a GO-CAM. The GO-CAM ShEx schema plus associated documentation is available from https://github.com/geneontology/go-shapes/.

### GO-CAM repository

Since their introduction two years ago, the number of GO-CAMs has increased to over 2838. 533 of these GO-CAMs contain at least one causal relation between activities, linking them into pathways. We are focusing effort on these pathway-oriented GO-CAMs, and expect this number to rise rapidly. A number of model organism databases, including members of the Alliance of Genome Resources (Alliance) (8) and Xenbase (9), are now generating GO-CAMs. In addition, we have initiated a phased project to migrate all standard manual GO annotations generated by Alliance member groups to GO-CAM starting with WormBase (10) and MGI (11). These standard GO annotations will be imported as gene-centric GO-CAMs, gathering all the curator-generated statements about gene function in a single model. The migrated standard GO annotations are also being checked against the ShEx schema to ensure compliance with the GO-CAM model. Going forward, these groups will curate GO-CAMs as their primary product. Users can now access the GO-CAM repository directly from the GO homepage.

## COLLABORATIONS

### SynGO: synapse biology

Led by the Center for Neurogenomics and Cognitive Research, Vrije Universiteit Amsterdam, the SynGO consortium is an international collaboration between 15 laboratories and GO to improve the curation of synapse biology. The project led to the revision of the GO ontology: the synapse branch of the GO Cellular Component aspect was expanded from 13 terms to 129 terms, and the synaptic signaling (GO:0099536) branch of the GO Biological Process aspect was expanded from 42 terms to 256 terms. In addition, SynGO produced over 6500 manual annotations to over 1100 distinct gene products supported by experimental results (12). More than half of these annotations are to mouse proteins, with most of the remainder to rat or human proteins, all of which were subsequently reviewed by the team at CNCR-VU and University College London.

### Rhea: catalytic activities

We initiated a project with the Rhea database (https://www.rhea-db.org) to represent catalytic activities in GO. Rhea is an expert-curated resource of biochemical reactions designed for the annotation of enzymes and metabolic networks and models. Rhea uses ChEBI (13) to precisely describe each reaction's participants and their chemical structures (14). Rhea is also the reference vocabulary for enzyme annotation in UniProtKB (15). As of September 2020, 4196 GO terms have been cross-referenced to Rhea using a combination of manual and computational methods, providing 47% coverage of catalytic activities in the GO. Such cross-references to Rhea enhance the consistency and interoperability of our representation of catalytic activities and expand the amount of annotations provided by GO, as those cross-references directly result in GO annotations for UniProt entries associated with those Rhea classes.

### GREEKC: DNA-binding transcription factors (dbTFs)

We have improved the ontology and annotations related to transcription factors, in collaboration with the GREEKC consortium (https://www.greekc.org/) (Gaudet *et al.*, Gene Ontology guidelines for transcription factor activity annotation, in preparation, and Lovering *et al.*, A GO catalog of human DNA-binding transcription factors, in preparation). GO now more clearly distinguishes the specific molecular functions of sequence-specific DNA-binding TFs, co-factors, and general TFs. This work is aimed at defining a gold-standard set of TF annotations for human and is being extended to other species, as a first pass via the review of more than 200 PANTHER families (3) by PAINT annotation (16).

## ANNOTATION EFFORTS

### Literature annotation highlights

Recent GO annotation efforts have been directed towards adding new annotations to increase the breadth and depth of the knowledgebase, and reviewing and improving existing annotations to reflect changing biological knowledge and updates to the ontology. Over the last two years, the number of GO annotations has increased by 10%, from 7 288 273 to 8 047 076, the annotated gene products by 26%, from 1 230 689 to 1 556 208 and the number of scientific articles annotated by 6%, from 151 236 to 160 660. About two-thirds of the increase in annotations and annotated entities are the result of PAINT annotations (see next section). These statistics correspond to net changes and underestimate the work of curators to add and remove evidence to support annotations of gene functions. In total, 13 166 new scientific articles were added over that period as evidence to support the annotation of gene functions and 3272 scientific articles were removed as a result of the review process mentioned in our last report (7). Articles were removed for two main reasons: (i) they were removed because the standard of evidence in the field has changed such that the evidence is not sufficient to support the annotation, or (ii) the annotations were found to be erroneous and (iii) because more recent experiments show the original interpretation was not correct. Table 1 shows the changes in GO annotations over the last two years for 11 well-studied organisms.

**Table 1.** Number of experimental (EXP) and phylogenetically inferred (PHYLO) annotations in the GO knowledgebase as of August 2020. Numbers in parentheses indicate the percentage net changes since August 2018. EXP corresponds to EXP, IDA, IEP, IGI, IMP and IPI evidence codes while PHYLO (curated phylogenetic inference using PAINT) corresponds to IBA, IRD, IKR and IMR evidence codes

	Protein binding EXP	Molecular function, EXP, excluding protein binding	Molecular function, PHYLO, excluding protein binding	Cellular component EXP	Cellular component PHYLO	Biological process EXP	Biological process PHYLO
Human	178 972 (+112%)	31 569 (+12%)	18 448 (+29%)	37 404 (+8%)	20 685 (+33%)	63 931 (+12%)	35 765 (+30%)
Mouse	14 233 (+9%)	15 118 (+5%)	19 792 (+26%)	26 010 (+5%)	21 277 (+31%)	76 777 (+6%)	38 304 (+26%)
Rat	4 564 (+5%)	11 253 (+3%)	19 672 (+26%)	16 385 (+4%)	21 226 (+33%)	31 434 (+3%)	37 126 (+26%)
Zebrafish	578 (+14%)	1 852 (+5%)	22 833 (+43%)	1 121 (+2%)	24 279 (+40%)	23 787 (+5%)	42 987 (+43%)
Drosophila	1 636 (+7%)	6 302 (+11%)	8 777 (+21%)	10 243 (+9%)	10 116 (+27%)	34 063 (+4%)	15 287 (+21%)
*C.elegans*	3 093 (+5%)	2 633 (+5%)	8 787 (+26%)	5 004 (+3%)	10 075 (+34%)	15 788 (+4%)	15 971 (+27%)
*D.discoideum*	853 (+23%)	1 168 (+8%)	5 597 (+26%)	2 421 (+11%)	6 356 (+32%)	4 700 (+7%)	9 329 (+23%)
*S.cerevisae*	176 (+5%)	8 697 (+2%)	4 566 (+9%)	10 552 (−1%)	5 770 (+22%)	24 108 (∼0%)	8 190 (+12%)
*S.pombe*	2 010 (−3%)	3 403 (+10%)	3 987 (+11%)	5 781 (+9%)	5 203 (+21%)	7 569 (+1%)	7 100 (+11%)
*A.thaliana*	16 151 (+13%)	9 868 (+16%)	12 767 (+8%)	27 526 (+7%)	13 879 (+15%)	30 597 (+6%)	19 101 (+5%)
*E.coli*	3 796 (+5%)	6 221 (+4%)	2 322 (+23%)	3 993 (−3%)	1 587 (+15%)	8 323 (+4%)	3 102 (+26%)

### Annotation review

GO annotation review is an ongoing process with the dual goals of improving annotation consistency and updating annotations to reflect current biological knowledge. While some level of annotation review is continually performed in response to isolated issues, over the past two years, we systematically focused our annotation reviews in two main areas: (i) annotation of gene products involved in transcription, with over 5000 annotations being reviewed, and (ii) use of relations in the annotation extensions (17) that provide context to standard GO annotations, with over 11 000 annotations being reviewed. Several thousand annotations have also been reviewed for smaller scale projects and individual tickets and error reports.

### Review of dubious annotation co-occurrences

The GO Term Matrix (18) can be used to query and visualize GO term co-annotation patterns. It has been used as part of a GO annotation quality control system to identify annotation errors and build annotation rules. These rules state that certain annotations cannot co-occur, for example few or no proteins play roles in both DNA replication and cytoskeleton organization. One annotation, to Pdgfb (platelet derived growth factor subunit B), a hormone, was identified as annotated to both terms. After review of the original paper, it was noticed that the DNA replication annotation was incorrect: DNA synthesis was used as a readout for a growth/proliferation phenotype, but as expected, Pdgfb does not itself play a role in DNA replication.

In certain cases, annotations are correct, but the incorrect co-annotation is due to an inference based on the ontology structure. For example, ‘protein citrullination’ (GO:0018101) used to be placed under ‘citrulline biosynthetic process’ (GO:0019240). The faulty relationship was removed since protein citrullination describes the modification of an amino acid residue in a protein by citrulline, and ‘citrulline biosynthetic process’ describes the synthesis of free citrulline.

### PAINT annotations

PAINT annotation is a GO curation effort to manually infer functions based on phylogenetic relationships (16). Since the last report in 2018, efforts have been made to both increase the number of annotations and improve their accuracy. Although GO is used to analyze genomes across the tree of life, a significant portion of our users work with vertebrates and in particular human. To better support these users, we focused our curation efforts over the last period on PANTHER families containing vertebrates and human gene products. As a consequence, about 2000 additional families encompassing 4200 human gene products were curated. In order to improve the accuracy of PAINT annotation, we focused on two main areas. The first was a review of previously curated families and updating of the annotations when new experimental annotations were added. The second was to implement a quality control mechanism to remove any taxon-constraint violations. As a result, significant progress has been made in generating new phylogenetically-inferred annotations (Table 1, PHYLO, columns 3, 5, 7)

### Statistics, quality control and GO releases

Statistics are now computed and tracked at each monthly GO release (http://geneontology.org/stats.html) and serve two purposes: (i) informing users of the changes in both the ontology and annotations and (ii) checking and validating the GO annotations of contributing groups before the GO publishes them in a public release. Although the GO pipeline is fully automated with numerous automated quality checks, we implemented an additional manual review step in which these statistics are evaluated by a quality assurance manager prior to any release. This additional check ensures the quality of our published data.

## ONTOLOGY REFINEMENTS

### Improving taxon constraints

Taxon constraints define valid taxa for GO terms that apply to some organisms but not to others. We addressed several issues that resulted in incomplete use of the GO taxon constraints, and have now removed these incorrect annotations (19). We worked with developers of external ontologies such as Uberon (20), the Cell Ontology (21), and the Fungal Anatomy Ontology to include taxon constraints within these ontologies, and to have these inherited in GO. For example, the cell ontology now constrains the term ‘leukocyte’ to vertebrates, and consequently terms in GO such as monocyte differentiation are inferred to be constrained to vertebrates.

### Leveraging inter-ontology inferences

GO makes extensive use of external ontologies, in particular Uberon (20), Cell Type Ontology (21), and ChEBI (13). Using external ontologies makes it easier to maintain the GO while leveraging domain experts in specific areas.

The ChEBI ontology, another major building block of GO, defines *roles* chemicals *may* have, e.g. ‘drug’ and ‘hormone’. GO, on the other hand, requires that the descendant of a term be *universally* true. This difference in practice resulted in incorrect logical inferences with respect to GO practices for a few specific cases, e.g. ‘ATP binding’ is not always a type of ‘drug binding’. Since ChEBI roles cannot always reliably be used to make inferences that are true in all contexts, they will no longer be used to make inferences about relationships between GO terms within the ontology.

### Maintaining the GO ontology up-to-date

**Figure 2.**
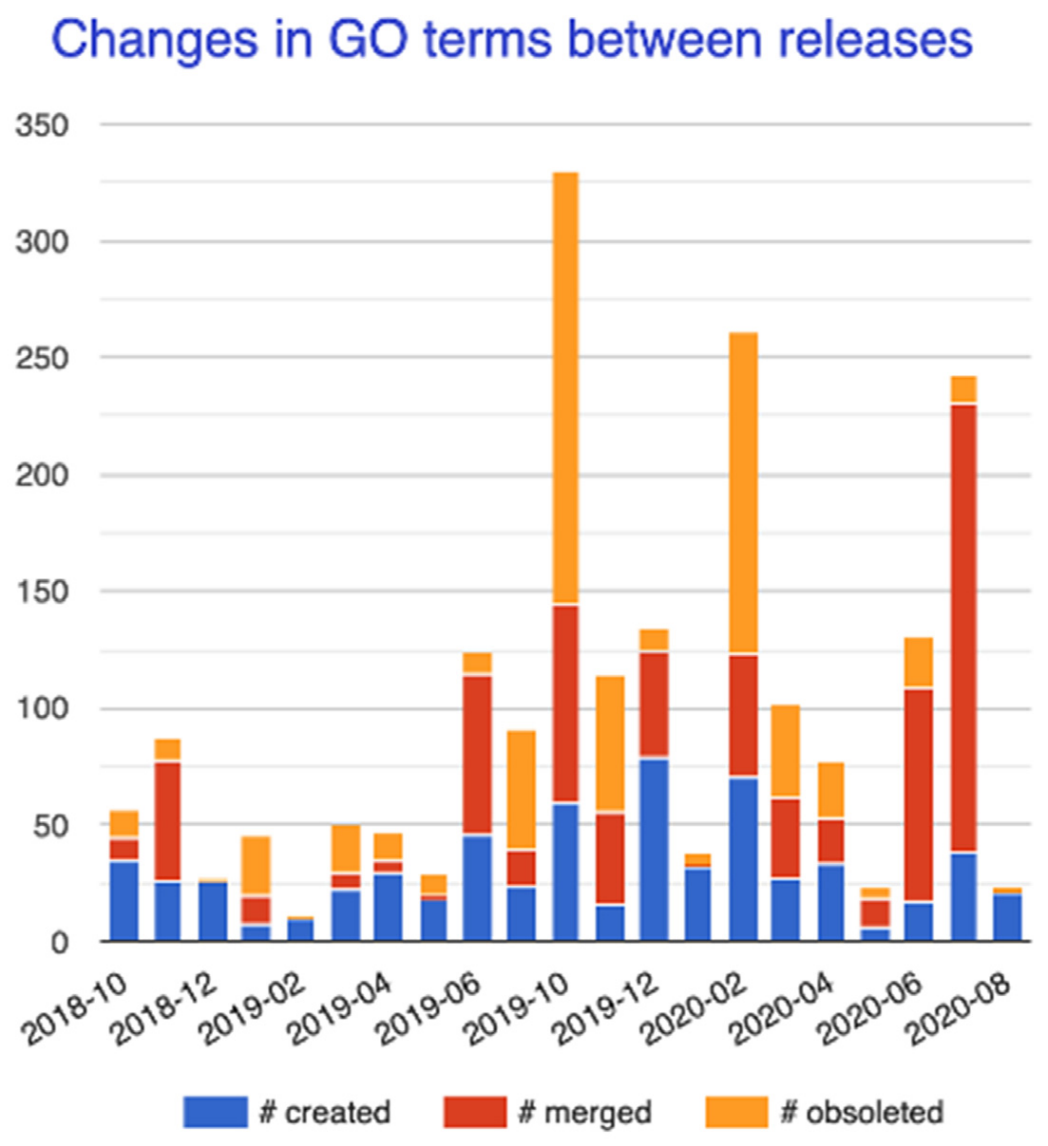
Changes in GO ontology statistics over the last two years. The stacked histograms detail the ontology efforts in creating, obsoleting and merging GO terms.

The GO ontology is under constant revision to address advances in biological knowledge, progresses in annotation practices and feedback from GO users. Through a collaborative process with domain experts both within and outside the GOC, we create new terms to describe new findings, and obsolete or merge terms that are unnecessary, incorrect or redundant. Over the last two years, we have created 672 new terms, obsoleted 661 terms, and merged 752 terms, resulting in a net decrease of 627 GO terms (Figure 2). The biggest change has been in biological process terms, due to refactoring the multi-organism part of the Biological Process branch and the obsoletion of combinatorial terms (e.g. GO:0100052, negative regulation of G1/S transition of mitotic cell cycle by transcription from RNA polymerase II promoter) made up of other GO terms which can now be linked using GO-CAM. Statistics are accessible at http://geneontology.org/stats.

## GO TOOLSET

### Noctua curation platform

**Figure 3.**
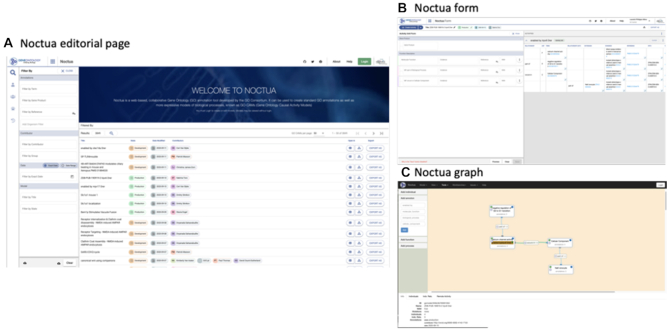
Noctua curation platform. (**A**) Editorial page where curators can browse and search, GO-CAMs. (**B**) Model edited in Noctua form, a simple form-based interface. (**C**) Noctua graph representation of the GO-CAM seen in (B). Both Noctua form and Noctua graph allow users to create and edit models.

Noctua (http://noctua.geneontology.org) is a set of curation tools that supports the collaborative creation and review of standard GO annotations and GO-CAMs (Figure 3). With over two years of feedback from GO curators, the consortium has created the **Noctua editorial page** to rapidly browse and search GO-CAMs, the **Noctua form** to ease the creation of GO-CAMs by employing a simple hierarchical form with predetermined attributes to fill, and the **Noctua graph** which helps visualize and edit the causal relationships between molecular activities. In addition, these tools have been designed to facilitate the incorporation of existing standard GO annotations into GO-CAMs to improve curation efficiency and maximize the reuse of existing annotations. We also integrated a system for the quick retrieval of bibliographic metadata from PubMed identifiers to provide curators with a more integrated and efficient workflow. Groups interested in curating GO-CAMs are welcome to contact us (see Helpdesk).

**Figure 4.**
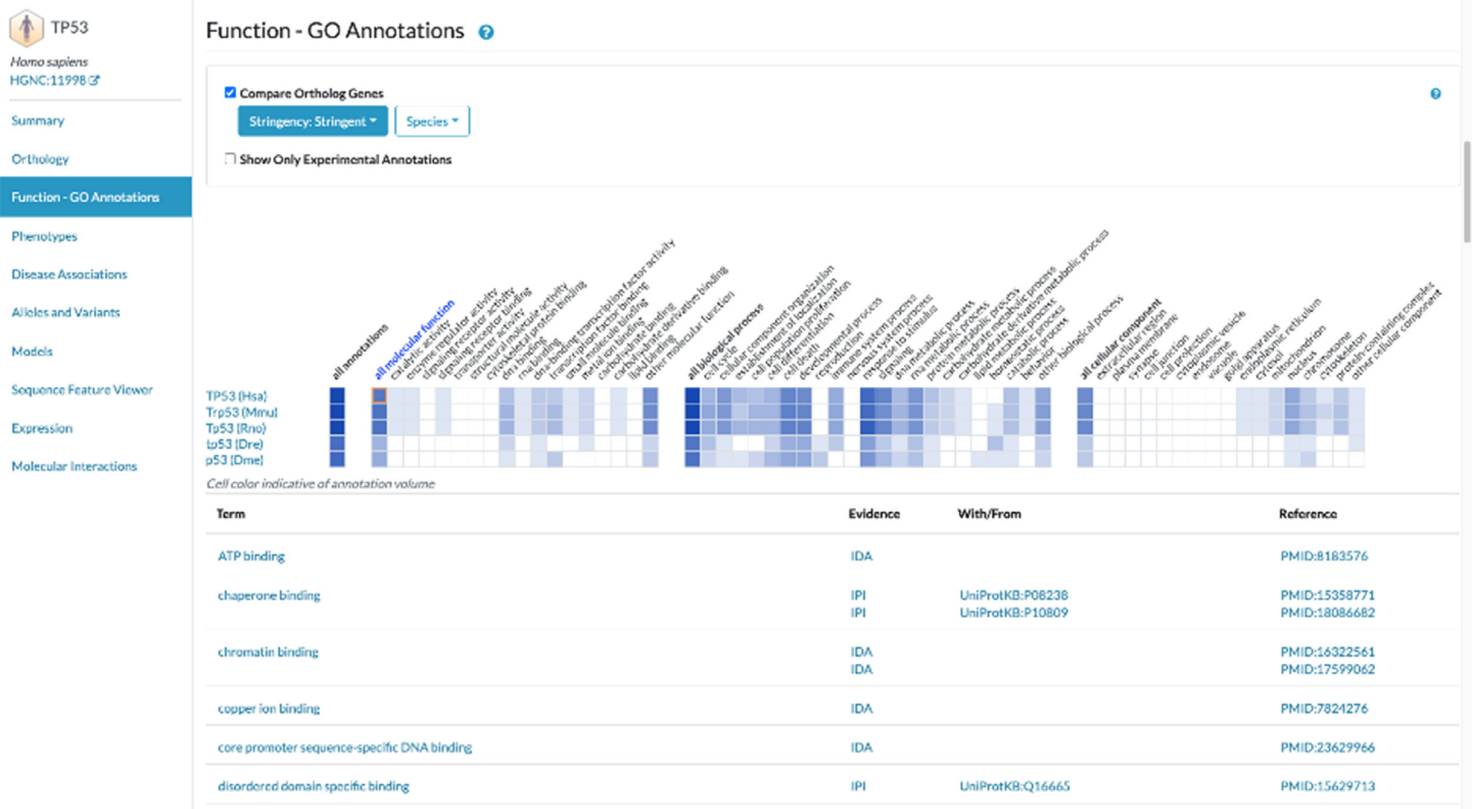
GO ribbon web component in the Alliance. This new version of the ribbon is fast, easy to integrate and now handles gene sets to quickly compare the function of several genes. In this example, the ribbon is used to highlight the differences between orthologs of TP53. Clicking on a high-level function (GO term) such as ‘cell death’ will open a table detailing all the annotations related to ‘cell death’, while clicking on ‘all molecular function’ will show all the available molecular function annotations for those genes.

### Querying GO-CAMs

The relative simplicity of standard GO annotations enabled their widespread use by allowing users to ask actionable biological questions (e.g. ‘what are the functions of a given gene’; ‘what are the scientific articles and the experimental evidence supporting that a gene has a certain function’). The rich semantic structure of GO-CAMs provided by OWL increases both the expressivity of the information captured by curators and gives the ability to perform more complex and targeted queries. The GO-CAM formalism thus enables queries such as ‘which genes’ activities are upstream or downstream of a given activity or process’ or ‘which genes’ activities directly positively regulate a given activity’. These advanced queries are designed with the SPARQL Query Language (https://www.w3.org/TR/sparql11-overview/) using the GO SPARQL endpoint http://sparql.geneontology.org. In addition, SPARQL enables the interoperability of RDF/OWL databases through federated queries (https://www.w3.org/TR/sparql11-federated-query/) (22). Federated queries allow, for instance, to query features of a GO-CAM and enrich these features with additional information available at other SPARQL endpoints such as UniProt (https://sparql.uniprot.org), identifiers.org (https://ebi.identifiers.org/services/sparql) and wikipathway (http://sparql.wikipathways.org).

### GO API

The GO ontology and annotations can be queried through the GO REST API (http://api.geneontology.org/api). This API follows the BioLink model (https://biolink.github.io/biolink-model/) whose purpose is to provide a common set of vocabulary and relationships to query biological data over multiple databases and fields in a consistent manner. A common interactive user interface, powered by swagger, is provided to help users design their queries. The GO API is hosted on the AWS cloud to ensure high availability and to provide a simple way to increase scalability on demand. It is currently used by the GO ribbon.

### GO ribbon

We previously reported the creation of a GO ribbon widget that provides a quick and interactive visual summary of the functions of a gene (7). The GO ribbon has been completely redesigned as a web component (Albou *et al.*, in preparation) to facilitate the integration in any website independently of the framework used, and to suit the needs of several projects such as the Alliance of Genome Resources (8) and the next iteration of the UniProt platform. In addition to a 100-x fold increase in speed to load these visual summaries, the ribbon now accepts a new generic data model able to represent any kind of association data (e.g. gene – go term; gene – disease; gene – expression, etc.). Because of its speed, the ribbon can now handle gene sets of several hundred and up to a thousand genes, thus providing a useful and quick summary of functions for any list of genes. That functionality was used by the Alliance of Genome Resources to show the conservation and divergence of functions between orthologs of a gene (Figure 4).

## GO WEBSITE

### GO website

In February 2019, we released an updated website for the GO, to help the 240 000 annual users find, browse, download and interact with GO data. We improved quick access to the documentation and tools such as GO Enrichment Analysis and AmiGO 2, the official tool for browsing standard GO annotations (Supplementary Figure S1). The new website is hosted on GitHub pages and uses the Jekyll framework for quick and versioned editing of our documentation. In addition, several forwarding URLs were created to provide backward compatibility to GO links previously referenced in articles and search engines such as Google. Useful links of the GO ecosystem are grouped in Table 2.

**Table 2.** Useful links to the GO ecosystem

	URL	Description
GO site	http://geneontology.org	Main entry to GO with quick access to search, enrichment, browse and downloads
GO releases	http://release.geneontology.org	Direct access to the GO releases’ files. This includes the GO archive reconstructed from 2005–2018 and the recent DOI GO releases
Latest GO release	http://current.geneontology.org	Convenient link to the latest GO release.
GO statistics	http://geneontology.org/stats	Statistics showing the changes in the GO ontology and annotations
AmiGO	http://amigo.geneontology.org	GO tool to browse GO ontology and annotations
GO Enrichment (Panther)	http://pantherdb.org	GO tool to perform Enrichment Analysis on gene sets and transcriptomic data
GO ribbon	http://www.geneontology.org/ribbon.html	GO tool to provide an interactive visual summary of the functions of a gene or gene sets
GO REST API	http://api.geneontology.org/api	Support common queries to GO
GO SPARQL	http://sparql.geneontology.org	Support common queries to GO-CAMs
Noctua curation platform	http://noctua.geneontology.org	Curation platform to browse, search, create and review GO-CAMs
Helpdesk	http://help.geneontology.org	Main entry point to ask questions and share feedback with GO
GO GitHub	https://github.com/geneontology	Access GO codes and ask more technical questions

### GO downloads

In 2018, we reported a new GO release pipeline which produces monthly releases of GO with a versioned DOI assigned by Zenodo (7,23). Quick access to download the ontology, the standard GO annotations and the GO-CAMs is available at http://geneontology.org/docs/downloads. All GO releases are additionally stored for long term archival at the CERN through Zenodo. Additional information on how to programmatically access those long term archives is detailed here: http://geneontology.org/docs/tools-guide/#programmatic-download-bdbag.

### GO historical archive

For releases that predate the first DOI release of GO in 2018, we have reconstructed an archive of 173 monthly releases dating from 2005 to 2018. The archive was generated using the data scattered across three legacy systems, namely the GO CVS, the GO SVN and the old product archive. Each of those systems was created at different times to serve different purposes and they were partially redundant, both in terms of the types of data they contained and in time frames (e.g. SVN was maintained from 2011 to 2018 while CVS was maintained from 2002 to 2018). Those three legacy systems will now be deprecated in favor of the new comprehensive GO archive covering the last 15 years of GO. To improve usability and simplify automated scripting, the GO archive is organized with the same folder hierarchy as the current GO releases and contains the ontology and annotations files from 2005–2018 as well as the legacy MySQL dumps of GO from 2005 to 2016. Future plans include producing the history of all GO term changes since 2005, as well as providing GO users with a GO enrichment tool able to analyze gene sets at different points in time. The ability to run GO enrichment analyses at different points in time would greatly help in the reproducibility of experiments (24) as well as to evaluate the impact of the revisions of GO.

## OUTREACH

### GO Helpdesk

The GO Helpdesk (http://help.geneontology.org/) is the general entry point for users having questions or feedback for GO. The Helpdesk provides several ways to connect with the GO consortium, from a simple email help@geneontology.org, to our Twitter feed (@news4go) as well as with a GitHub repository (https://github.com/geneontology/helpdesk). We encourage users to create tickets on the GitHub repository so that questions and feedback can be shared with and help the broader GO community. General announcements such as data releases and obsoletion notices are also posted on GitHub (https://github.com/geneontology/go-announcements), sent out to the subscribers of our go-friends mailing list (go-friends@lists.stanford.edu) and on our Twitter and Facebook pages. Those channels are also used to announce users meetings where we inform on the latest advances, provide training on various aspects of GO and get more direct feedback from our users. A compendium of the Frequently Asked Questions are directly accessible from the Help menu of the GO website (http://geneontology.org/docs/faq).

## Supplementary Material

gkaa1113_Supplemental_File

## APPENDIX

Authors

Berkeley Bioinformatics Open-Source Projects (BBOP), Environmental Genomics and Systems Biology Division, Lawrence Berkeley National Laboratory (Berkeley, CA, USA): Seth Carbon, Eric Douglass, Benjamin M. Good, Deepak R. Unni, Nomi L. Harris, Christopher J. Mungall; dictyBase, Northwestern University (Chicago, IL, USA): Siddartha Basu, Rex L. Chisholm, Robert J. Dodson, Eric Hartline, Petra Fey; Division of Bioinformatics, Department of Preventive Medicine, University of Southern California (Los Angeles, CA, USA): Paul D Thomas, Laurent-Philippe Albou, Dustin Ebert, Michael J Kesling, Huaiyu Mi, Anushya Muruganujan, Xiaosong Huang, Tremayne Mushayahama; EcoliWiki, Departments of Biology and Biochemistry and Biophysics, Texas A&M University (College Station, TX, USA): Sandra A. LaBonte, Deborah A. Siegele; FlyBase, Department of Physiology, Development and Neuroscience, University of Cambridge (Cambridge, UK): Giulia Antonazzo, Helen Attrill, Nick H. Brown, Phani Garapati, Steven J. Marygold, Vitor Trovisco; FlyBase, The Biological Laboratories, Harvard University (Cambridge, USA): Gil dos Santos, Kathleen Falls, Christopher Tabone, Pinglei Zhou; FlyBase, Department of Biology, Indiana University, (Bloomington, USA): Joshua L. Goodman, Victor B. Strelets, Jim Thurmond; GO-EMBL-EBI (Hinxton, UK): Penelope Garmiri, Rizwan Ishtiaq, Milagros Rodríguez-López, Gene Regulation Consortium (GRECO), Norwegian University of Science and Technology (Trondheim, Norway): Marcio L. Acencio, Martin Kuiper, Astrid Lægreid; Gene Regulation Consortium (GRECO), Radboud University (Nijmegen, The Netherlands): Colin Logie; Functional Gene Annotation, Institute of Cardiovascular Science, University College London (London, UK): Ruth C. Lovering, Barbara Kramarz, Shirin C.C. Saverimuttu, Sandra M. Pinheiro, Heather Gunn, Renzhi Su, Katherine E. Thurlow; Institute for Genome Sciences, University of Maryland School of Medicine (Baltimore, MD, USA): Marcus Chibucos, Michelle Giglio, Suvarna Nadendla, James Munro, Rebecca Jackson; IntAct/Complex Portal, EMBL-EBI (Hinxton, UK): Margaret J. Duesbury, Noemi Del-Toro, Birgit H.M. Meldal, Kalpana Paneerselvam, Livia Perfetto, Pablo Porras, Sandra Orchard, Anjali Shrivastava, InterPro, EMBL-EBI (Hinxton, UK): Hsin-Yu Chang, Robert Daniel Finn, Alexander Lawson Mitchell, Neil David Rawlings, Lorna Richardson, Amaia Sangrador-Vegas; Mouse Genome Informatics, The Jackson Laboratory (Bar Harbor, ME, USA): Judith A. Blake, Karen R. Christie, Mary E. Dolan, Harold J. Drabkin, David P. Hill, Li Ni, Dmitry M. Sitnikov; PomBase, University of Cambridge (Cambridge, UK): Midori A. Harris, Stephen G. Oliver, Kim Rutherford, Valerie Wood; PomBase, The Francis Crick Institute (London, UK): Jaqueline Hayles; PomBase, University College London (London, UK): Jürg Bähler; RGD, Medical College of Wisconsin (Milwaukee, WI, USA): Elizabeth R. Bolton, Jeffery L. De Pons, Melinda R. Dwinell, G.Thomas Hayman, Mary L. Kaldunski, Anne E. Kwitek, Stanley J. F. Laulederkind, Cody Plasterer, Marek A. Tutaj, Mahima Vedi, Shur-Jen Wang; Reactome, Department of Biochemistry & Molecular Pharmacology, NYU Grossman School of Medicine (New York, NY, USA): Peter D’Eustachio, Lisa Matthews; Renaissance Computing Institute, University of North Carolina (Chapel Hill, NC, USA): James P. Balhoff; SGD, Department of Genetics, Stanford University (Stanford, CA, USA): Suzi A. Aleksander, Michael J. Alexander, J. Michael Cherry, Stacia R. Engel, Felix Gondwe, Kalpana Karra, Stuart R. Miyasato, Robert S. Nash, Matt Simison, Marek S. Skrzypek, Shuai Weng, Edith D. Wong; SIB Swiss Institute of Bioinformatics (Geneva, Switzerland): Marc Feuermann, Pascale Gaudet, Anne Morgat; The Arabidopsis Information Resource (TAIR), Phoenix Bioinformatics (Fremont, CA, USA): Erica Bakker, Tanya Z. Berardini, Leonore Reiser, Shabari Subramaniam, Eva Huala; UniProt: EMBL-EBI (Hinxton, UK), SIB Swiss Institute of Bioinformatics (SIB) (Geneva, Switzerland), and Protein Information Resource (PIR) (Washington, DC, USA and Newark, DE, USA): Cecilia N. Arighi, Andrea Auchincloss, Kristian Axelsen, Ghislaine Argoud-Puy, Alex Bateman, Marie-Claude Blatter, Emmanuel Boutet, Emily Bowler, Lionel Breuza, Alan Bridge, Ramona Britto, Hema Bye-A-Jee, Cristina Casals Casas, Elisabeth Coudert, Paul Denny, Anne Estreicher, Maria Livia Famiglietti, George Georghiou, Arnaud Gos, Nadine Gruaz-Gumowski, Emma Hatton-Ellis, Chantal Hulo, Alexandr Ignatchenko, Florence Jungo, Kati Laiho, Philippe Le Mercier, Damien Lieberherr, Antonia Lock, Yvonne Lussi, Alistair MacDougall, Michele Magrane, Maria J. Martin, Patrick Masson, Darren A. Natale, Nevila Hyka-Nouspikel, Sandra Orchard, Ivo Pedruzzi, Lucille Pourcel, Sylvain Poux, Sangya Pundir, Catherine Rivoire, Elena Speretta, Shyamala Sundaram, Nidhi Tyagi, Kate Warner, Rossana Zaru, Cathy H. Wu; University at Buffalo, Department of Biomedical Informatics (Buffalo, NY, USA): Alexander D. Diehl; WormBase: California Institute of Technology (Pasadena, CA, USA), Juancarlos N. Chan, Christian Grove, Raymond Y.N. Lee, Hans-Michael Muller, Daniela Raciti, Kimberly Van Auken, Paul W. Sternberg, Wellcome Trust Sanger Institute (Hinxton, UK), Matthew Berriman, EBI (Hinxton, UK), Michael Paulini, Kevin Howe, and Ontario Institute for Cancer Research (Toronto, Canada):Sibyl Gao, Adam Wright, Lincoln Stein,; ZFIN, University of Oregon (Eugene, OR, USA): Douglas G Howe, Sabrina Toro, Monte Westerfield. Gramene, Planteome and Oregon State University (Corvallis, OR, USA): Pankaj Jaiswal, Laurel Cooper, Justin Elser.
