# Supplementary material for: The Gene Ontology resource: enriching a GOld mine
Source: Nucleic Acids Res. 2020 Dec 8;49(D1):D325–34. doi: 10.1093/nar/gkaa1113 (PMC7779012; doi:10.1093/nar/gkaa1113)
Supplement: gkaa1113_Supplemental_File [file gkaa1113_supplemental_file.pdf]

SUPPLEMENTARY

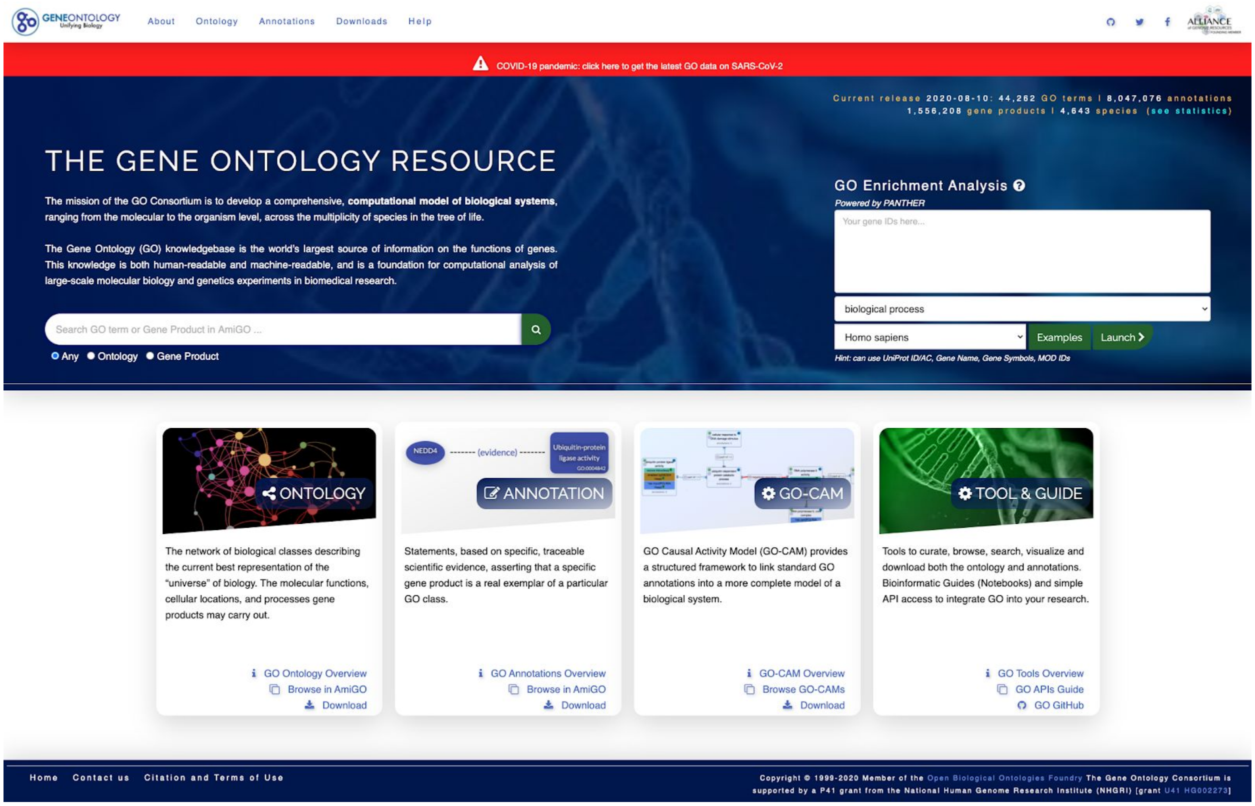

Figure 1. The new GO website emphasizes the three components of GO (ontology, standard GO annotations, GO-CAMs) and the tools provided by the consortium. Each card provides a quick access to the documentation ("Overview"), data ("Browse") and downloads.
